# Personality factors and cognitive functioning in elderly with Parkinson's disease

**DOI:** 10.1590/1980-57642018dn12-010007

**Published:** 2018

**Authors:** Neusa Maria de Oliveira Chardosim, Camila Rosa Oliveira, Manuela Polidoro Lima, Marianne Farina, Valéria Gonzatti, Dalton Breno Costa, Aline Sória Pereira, Luis Henrique Paloski, Tatiana Quarti Irigaray, Irani Iracema de Lima Argimon

**Affiliations:** 1 Pontifícia Universidade Católica do Rio Grande do Sul (PUCRS), Porto Alegre, RS, Brazil; 2 Faculdade Meridional (IMED), Passo Fundo RS, Brazil; 3 Universidade Federal de Ciências da Saúde de Porto Alegre (UFCSPA), Porto Alegre, RS, Brazil

**Keywords:** Parkinson's disease, personality factors, cognitive decline, quality of life, doença de Parkinson, fatores de personalidade, declínio cognitivo, qualidade de vida

## Abstract

Parkinson's disease (PD) is a chronic and progressive neurological disease, resulting from cell degeneration in the substantia nigra, responsible for the production of dopamine.

**Objective:**

This study aimed to characterize the cognitive functioning, personality factors and prevalence of depressive and anxiety symptoms in individuals with Parkinson's disease (PD). Furthermore, this study sought to analyze whether personality factors were predictors of cognitive functioning.

**Methods:**

The sample consisted of 30 elderly with PD. Participants completed a sociodemographic data sheet, the NEO-FFI-R (Five Factor Inventory NEO Revised), the Montreal Cognitive Assessment, the Beta-III, the phonemic verbal fluency test and semantics (Animals), the digits span subtest of the Wechsler Intelligence Scale for Adults and the Boston Naming Test and the word list of the CERAD battery, the Geriatric Depression Scale and the Beck Anxiety Inventory.

**Results:**

The elderly with PD presented impairment in verbal episodic memory and executive functions. Most of the participants demonstrated low levels of neuroticism. The extraversion factor was positively correlated with executive functions and the openness to experience factor was positively correlated with verbal episodic memory. It was concluded that the elderly with PD presented memory and executive function impairments. The factor that most contributed to performance of the elderly with PD on memory and executive function tasks was the extraversion factor.

## RESUMO

A doença de Parkinson (PD) é uma doença neurológica crônica e progressiva, resultante da degeneração celular na substância negra, responsável pela produção de dopamina.

**Objetivo:**

Este estudo teve como objetivo caracterizar o funcionamento cognitivo, fatores de personalidade e prevalência de sintomas de depressão e ansiedade em indivíduos com doença de Parkinson (DP). Além disso, este estudo procurou analisar se os fatores de personalidade eram preditores de funcionamento cognitivo.

**Métodos:**

A amostra foi constituída por 30 idosos com DP. Os participantes responderam a uma ficha de dados sociodemográficos, o NEO-FFI-R (Inventário de Cinco Fatores NEO Revisado), o Montreal Cognitive Assessment (MoCA), o Beta III, o Teste de Fluência Verbal Semântica (Categoria Animais), o Subteste Dígitos da Escala de Inteligência Wechsler para adultos, o teste de nomeação de Boston da bateria CERAD, a Escala de Depressão Geriátrica e o Inventário de Ansiedade de Beck.

**Resultados:**

Os idosos com DP apresentaram comprometimento nas funções de memória episódica verbal e funções executivas. A maioria dos participantes demonstrou baixos níveis de neuroticismo. O fator de extroversão foi positivamente correlacionado com as funções executivas e o fator de abertura à experiência foi positivamente correlacionado com a memória episódica verbal.

**Conclusão:**

Conclui-se que idosos com DP apresentam comprometimento na memória e nas funções executivas. O fator que mais contribuiu para o desempenho dos idosos com DP em tarefas de memória e funções executivas foi o fator extroversão.

Parkinson's disease (PD) is a chronic and progressive neurological disease, resulting from cell degeneration in the substantia nigra, responsible for the production of dopamine.^1^ The disease affects 1% of the world's population over 65 years old, totaling approximately six million people.^2^ The annual prevalence of the disease is around 160 cases per 100,000 population and its incidence is 4-21 cases per 100,000 population.^3^ According to 2000 Census data, the life expectancy for the Brazilian population over 65 years increased by 21%. Therefore, it is estimated that 200,000 individuals will present PD, increasing the number of affected individuals in Brazil.^4^^,^ 5

PD is characterized by motor symptoms, such as resting tremor, muscular rigidity, postural instability, balance and gait disorders and other manifestations.^6^ However, non-motor symptoms also occur during the course of the disease, such as changes in smell and disturbances in sleep,^7^ which may promote physical and mental disability. In addition, patients may present cognitive deficits, as well as anxiety and depression symptoms, which cause significant impairments in quality of life.^8^

According to Radanovic, Stella, and Forlenza,^9^ cognitive deficits may occur in the early stages of PD, manifested by alterations in episodic memory, language and thought. The most common cognitive changes in PD are problems of learning, memory, visuospatial processes and executive functions.^10^^,^ 11 Deficits in executive functions are primarily associated with frontal lobe pathology, specifically due to the lack of dopamine in PD.^12^^,^ 13

Janvin et al.^14^ found that over 50% of PD patients without dementia, demonstrated some cognitive alterations, and 20% had mainly memory deficits. In addition, 30% of patients suffer from executive dysfunctions. According to Galhardo, Amaral and Vieira,^15^ changes in mnemonic, visuospatial, language and executive functioning occurred in individuals with PD.

According to studies, cognitive performance in elderly may be influenced by personality factors.^16^^-^ 18 Personality may be understood as a continuum, in which healthy aspects such as autonomy, efficacy and adaptation to the environment are present, besides pathological factors, where the individual exhibits difficulties in adapting to specific contexts.^19^

There are countless models for studying personality. In the present study, the Big Five Factors model (BFF) was chosen, which comprises and characterizes personality for different factors: a) Neuroticism, which refers to the tension manifested in experiences of anxiety, anger and depression; b) Extraversion, which is related to sociability and vivacity; c) Openness to experience, which is associated with creativity, intellectual curiosity and need for variety; d) Agreeableness or socialization, which corresponds to the experiences of trust, altruism and sympathy; and e) Conscientiousness or achievement, which refers to the fulfilment of goals and values.^17^^,^ 20

Among the BFF, high scores in neuroticism have been highlighted^16^^,^ 18 and low scores in openness to experience lead to worse cognitive functioning of elderly individuals.^16^^,^ 21 Elevated neuroticism scores may also represent a risk factor for the development of mild cognitive decline (MCD) and dementia in the elderly.^16^^,^ 18 ^,^ 20 Sutin et al.^22^ showed that high levels of extraversion and low neuroticism are associated with better performance of patients on tasks of episodic memory. According to Booth et al.^23^ high levels of openness to experience are associated with better performance in verbal memory and general cognitive ability.

In the literature, few studies have investigated personality factors in patients with PD. Studies have found that individuals with this pathology had a "Parkinsonian Personality", presenting specific aspects common to patients with PD. Individuals with PD tend to be more nervous, insecure, pessimistic, reserved, less exploratory and curious and dependent when compared to healthy elderly. However, it is believed that the Parkinsonian personality profile may be related to the recurrent existence of depression concomitant with PD.^10^^,^ 24

The study of the relationship between personality factors and cognitive functioning in elderly with PD is recent. Most studies investigate these variables in isolation or in healthy elderly individuals. Thus, the present study aimed to: a) characterize cognitive functioning, personality factors and prevalence of depressive and anxiety symptoms in patients with PD; b) verify relationships between personality factors, sociodemographic characteristics (age and education), depressive and anxiety symptoms and cognitive performance; c) analyze whether personality factors are predictors of cognitive functioning in elderly with PD.

## METHODS

### Design

A cross-sectional, correlational, exploratory study was conducted.

### Participants

A total of 30 elderly with PD participated in this study, recruited from the Parkinson's Association of Rio Grande do Sul (APARS) and the Ambulatory Movement Disorders unit of the São Lucas Hospital of Porto Alegre, Rio Grande do Sul, Brazil. All participants were diagnosed with PD by a neurologist and were using medication to control the disease. One participant was excluded for having a score on MoCA suggestive of dementia.

### Instruments

*Sociodemographic Data Sheet:* which included the variables age, sex and education.

*Five Factor Inventory NEO Revised - NEO-FFI-R (reduced version):* Evaluates the five personality factors: Neuroticism, extraversion, openness, agreeableness and conscientiousness. The Cronbach's alpha coefficient of the instrument varies from 0.70 to 0.83 on the different factors.^25^^,^ 26

*Digit Span Subtest of The Wechsler Adult Intelligence Scale (WAIS-III):* This measures working memory, the extent of concentrated verbal attention, immediate memory retention and the ability for reversibility. Normative data for elderly people are available.^27^^,^ 28

*Montreal Cognitive Assessment - MoCA:* This is an instrument for screening dementia. The test totals 30 points where a score ≥26 points indicates normality, and <25 points indicates MCI.^29^ The MoCA has a Cronbach's alpha of 0.83, high internal consistency, 90% sensitivity in the recognition of MCI, 100% sensitivity in the recognition of Alzheimer's disease, and specificity of 87%.^30^ In this study, individuals with dementia scoring a total of less than 21 points were excluded.

*Phonemic Verbal Fluency Test - FAS:* This measures phonemic verbal fluency with a sensitive measure of executive functions.^31^ In the study by Machado et al.,^32^ normative data were provided for the elderly population (60-93 years old). The FAS has a Cronbach's alpha of 0.83.^33^

*Semantic Verbal Fluency Test - Animals Category:* This test evaluates the processing of executive functions, especially those assessing the ability to organize thought and the strategies utilized for word searching.^31^ Normative data for the elderly population were established by Brucki et al.^34^

*Word list subtest and Boston Naming Test of the CERAD Battery*: The word list evaluates verbal episodic memory, besides immediate and delayed recall and recognition. The Boston naming test (BNT) is considered a language test and evaluates the ability of naming and visual perception.^35^

*Matrix Reasoning of the BETA III:* Evaluates the processing of visual information and abstract reasoning and is a measure of general intelligence.^36^

*Beck Anxiety Inventory - BAI:* Evaluates the intensity of anxiety symptoms. The summed items result in a total score ranging from zero to 63 points.^37^

*Geriatric Depression Scale (GDS-15):* This is a measure utilized to identify and quantify depressive symptoms in the elderly. The lowest possible score is zero and the highest is 15.^38^

*Modified Hoehn Yahr Scale*: This is a measure of the PD stages describing instability, rigidity, tremor, and bradykinesia.^39^

### Data collection

First, this study was approved by the Research Ethics Committee of *Pontifícia Universidade Católica do Rio Grande do Sul* under the number CAAE 51228015.5.0000.5336. After approval, contact was made with the Parkinson's Association of Rio Grande do Sul (*APARS*) and the Ambulatory Movement Disorders of the *Hospital São Lucas de Porto Alegre, Rio Grande do Sul, Brazil,* where participants were invited to take part in the study. Participants who agreed to take part completed and signed the Free and Informed Consent Form. The participants then completed the evaluation instruments which were applied in the following order: Sociodemographic data sheet, MoCA, digits span, battery word list CERAD (recall), Matrix Reasoning - BETA III; Word list (recall), word list (recognition), phonemic and semantic verbal fluency, Boston naming test, GDS 15, BAI and NEO-FFI-R. Each participant was evaluated during a single session lasting three hours.

### Data analysis

The data were analyzed using the statistical package SPSS, version 23 for Windows. Descriptive analyses (mean, standard deviation and percentages) and inferential statistics were utilized. Data distribution was investigated by the Kolmogorov-Smirnov test. The classification of performance on the instruments was performed by calculating the *z* score (digits span subtest, phonemic and semantic verbal fluency tasks, list of words on CERAD battery and Boston Naming Test) or specific cut-off points (MoCA, Beta III, GDS-15, BAI, NEO-FFI-R). Pearson's correlation was utilized to verify associations between personality characteristics, age, education, depressive and anxiety symptoms. Partial correlations, controlling for the effects of age, education, and depressive and anxiety symptoms, were utilized to determine the association between cognitive performance and personality characteristics. Based on the results of the correlations, linear regression analysis was performed using the entry method to verify the impact of personality factors on cognitive performance. The results were considered significant if *p* <0.05.

## RESULTS

A total of 30 individuals, age range 60-86 years, mean age 68.97 years (SD = 6.35), including 17 (56%) women participated in this study. Disease duration ranged from 1 to 18 years, mean 7.1 years (SD 5.64). Education ranged from 4 to 20 years, mean 12.27 years (SD = 5.19). Regarding depressive and anxiety symptoms, mean score obtained on the GDS-15 was 5.17 (SD = 3.20) and 43% (n = 13) of participants had scores suggestive of significant depressive symptoms. Mean score on the BAI was 14.77 (SD = 11.70) and 27% (n = 8) of participants exhibited moderate-to-severe anxiety symptoms. According to the Modified Hoehn Yahr Scale,^39^ most participants were in the early stages, with 15 (50%) classified as Stage 1 and 6 (20%) patients as Stage 2. Regarding cognitive performance, the classification of participant performance is given in Table 1.

**Table 1:** Mean, Standard Deviation (SD) and Performance Classification on Cognitive Instruments

	Descriptive				Classification	
	M	SD	Range		Normal n (%)	Deficit n (%)
DO Digits - Score	6.93	1.87	4-11		27 (90)	3 (10)
IO Digits - Score	4.23	1.55	2-7		30 (100)	0 (0)
CERAD-WL - Immediate recall	14.57	4.97	3-23		21 (70)	9 (30)
CERAD-WL - Delayed recall	2.97	2.16	0-8		22 (73)	8 (27)
CERAD-WL - Recognition	7.40	2.54	1-10		20 (67)	10 (33)
MR - BETA-III	11.23	4.20	5-5		24 (80)	6 (20)
VF - FAS	29.47	12.92	7-70		30 (100)	0 (0)
VF - Animals	12.97	4.35	4-22		28 (93)	2 (7)
BNT	13.50	1.53	9-15		29 (97)	1 (3)

M: Mean; SD: Standard Deviation; DO: Direct order; IO: Indirect order; CERAD-WL: Consortium to Establish a Registry for Alzheimer's Disease; -Word List Subtest; MR: Matrix Reasoning of BETA III; VF: Verbal Fluency; BNT: Boston Naming Test.

On the evaluation of cognitive performance, the percentage of participants whose scores were suggestive of deficits ranged from 0% to 33%. The greatest deficits were found for verbal episodic memory (recognition). On the Digits span (indirect order) and verbal fluency (phonemic modality) tasks, which evaluate components of executive functions, all participants had satisfactory performance according to age and education. The means and standard deviations for the personality factors are depicted in Table 2.

**Table 2:** Mean, Standard Deviation (SD) and Classification of Personality Factors.

	Descriptive				Classification		
	M	SD	Range		Average n (%)	High n (%)	High n (%)
NEO FFI-RP - Neuroticism	23.60	9.17	5-43		12 (40)	10 (33)	8 (27)
NEO FFI-RP - Extraversion	25.17	10.84	1-40		15 (50)	6 (20)	9 (30)
NEO FFI-RP - Openness	29.53	6.02	16-39		15 (50)	11 (36)	4 (14)
NEO FFI-RP - Agreeableness	34.70	6.28	21-46		7 (23)	9 (30)	14 (47)
NEO FFI-RP - Conscientiousness	34.37	5.61	23-45		4 (14)	13 (43)	13 (43)

M: Mean; SD: Standard Deviation; NEO FFI-RP: Five-Factor Inventory.

Regarding the personality factors, there was a higher frequency of low scores for extraversion and openness, while agreeableness and conscientiousness factors demonstrated a higher frequency of high scores classified. The results of the correlation analyses (Pearson and partial) between personality factors, age, education, cognitive performance and anxiety and depression symptoms are shown in Table 3.

**Table 3:** Correlations between the Factors of Personality, Age, Education, Depressive and Anxiety Symptoms and Cognitive Performance.

	NEO-FFI-R				
	Neuroticism	Extraversion	Openness	Agreeableness	Conscientiousness
Age	-0.291	-0.264	-0.142	0.209	-0.033
Education	0.165	-0.075	0.171	-0.168	-0.263
GDS-15	0.660***	-0.655***	-0.322	-0.504**	-0.396*
BAI	0.632**	-0.517**	-0.177	-0.433*	-0.326
DO Digits - Score	0.100	-0.119	-0.021	-0.118	0.107
IO Digits - Score	0.029	0.244	-0.070	-0.046	0.138
CERAD-WL - Immediate recall	0.044	0.377	0.190	-0.151	0.082
CERAD-WL - Delayed recall	0.194	0.373	0.407*	-0.088	-0.092
CERAD-WL - Recognition	0.086	0.289	-0.152	-0.156	-0.007
MR - BETA-III	-0.405*	0.272	-0.023	0.275	-0.047
VF - FAS	0.329	0.436*	0.291	-0.139	0.004
VF - Animals	0.155	0.361	-0.042	-0.022	-0.230
BNT	0.243	0.095	0.276	0.184	0.171

GDS-15: Short Geriatric Depression Scale; BAI: Beck Anxiety Inventory; DO: Direct order; IO: Indirect order; CERAD-WL: Consortium to Establish a Registry for Alzheimer's Disease - Word List subtest; MR: Matrix Reasoning; VF: Verbal Fluency; BNT: Boston Naming Test.

* p <0.05;

** p ≤0.01;

*** p ≤0.001;

Associations between personality characteristics, age, education, and anxiety and depression symptoms were performed using Pearson's correlation, whereas associations between personality factors and cognitive performance were verified through partial correlation, controlling for the effect of age, education, and depression and anxiety symptoms.

In relation to depressive symptoms on the GDS-15, a significant, strong positive correlation with the factor neuroticism, and a strong, negative correlation with extraversion, was found. Moderate and negative associations with agreeableness and conscientiousness factors were found. BAI scores also demonstrated significant, positive, strong correlations with neuroticism, and moderate and negative correlations with extraversion and agreeableness. Thus, the higher the level of neuroticism, the greater the depressive and anxiety symptoms.

With regard to cognitive performance, the neuroticism factor correlated significantly, negatively and moderately, with the result on the Beta-III, indicating that the higher the level of neuroticism, the lower the performance on tasks of abstract reasoning. The extraversion factor showed a moderate positive association with the phonemic modality of the verbal fluency task. Therefore, the greater the extraversion (sociability) ability, the better the results for general cognitive capacity and executive functions on the phonemic verbal fluency task. The openness factor showed only a moderate, positive, significant association with delayed recall on the CERAD Word List subtest. Thus, in relation to this sample, the more open to new experiences, the better the performance of episodic verbal memory (delayed recall). The other personality factors did not show significant correlations with the variables investigated. The linear regression analyses are depicted in Table 4.

**Table 4:** Regression Analysis Models.

	B ± SE	b	t	R^2^	F	p
CERAD-WL - Immediate recall						
NEO FFI-RP - Extraversion	0.251 ± 0.072	0.548	3.465	0.275	12.007	0.002
CERAD-WL - Delayed recall						
NEO FFI-RP - Extraversion	0.056 ± 0.035	0.281	1.585	0.238	5.531	0.010
NEO FFI-RP - Openness	0.129 ± 0.063	0.360	2.034			
CERAD-WL - Recognition						
NEO FFI-RP - Extraversion	0.115 ± 0.039	0.491	2.982	0.214	8.890	0.006
VF - FAS						
NEO FFI-RP - Extraversion	0.479 ± 0.206	0.402	2.322	0.132	5.393	0.028
VF - Animals						
NEO FFI-RP - Extraversion	0.179 ± 0.068	0.445	2.633	0.170	6.933	0.014

Unstandardized regression coefficient; SE: standard error; β: standardized regression coefficient; R2: squared multiple correlation coefficient; NEO FFI-RP: Five-Factor Inventory; CERAD-WL: Consortium to Establish a Registry for Alzheimer's Disease- Word List subtest; VF: verbal fluency.

According to the regression models, the extraversion factor contributed approximately 28% in immediate evocation memory, 21% in recognition memory, 13% in phonemic verbal fluency and 17% in semantic verbal fluency. Overall, the extraversion and openness factors contributed approximately 24% in the episodic memory scores for delayed recall.

## DISCUSSION

The results of this study demonstrated that the participants with PD had cognitive impairment, mainly in the functions of episodic memory (immediate recall, delayed recall and recognition) and executive functions (abstract reasoning and problem-solving). This result corroborates findings from previous studies.^40^^,^ 41

Memory deficits are among the most frequent changes observed in PD, characterized by difficulty recalling recently learned verbal information or impaired ability to use semantic coding effectively.^40^ From the onset of PD, changes in executive functions are observed, which are independent of impairments in other cognitive functions.^41^

Regarding the prevalence of depression and anxiety symptoms in this study, 43% of the sample had scores suggestive of depressive symptoms and 27% suggestive of anxiety symptoms. The rates found were high due to the fact that all of the PD patients were in use of medication and under medical supervision. According to previous studies, symptoms of depression and anxiety are common in PD and often coexist.^42^^,^ 43 The manifestation of depressive and anxiety symptoms may be in response to the diagnosis of the disease, given the patient is aware that PD is a chronic and neurodegenerative disease; or may be a consequence of neurological degeneration.^10^^,^ 36

Previous studies have reported similar findings of depressive and anxiety symptoms in patients with PD. Rocha^40^ found a prevalence of 47% of mild or moderate depressive symptoms in individuals with PD. Another study found a prevalence of anxiety symptoms of 22% in PD patients, where 8.6% of the sample presented anxiety symptoms coexisting with depressive symptoms.^44^

Regarding the relationship between depression and anxiety symptoms and personality factors in the present study, a higher level of neuroticism correlated with greater depressive and anxiety symptoms. These findings corroborate the results of other studies. Farina, Argimon and Irigaray^45^ also found that depressive symptoms exhibited a positive relationship with the neuroticism factor, and a negative association with the factors extraversion and conscientiousness, as well as a positive association between anxiety symptoms and the neuroticism factor in healthy elderly individuals. In the study by Koorevaar et al.,^46^ severe symptoms of depression were directly associated with the neuroticism factor and inversely with the extraversion and conscientiousness factors in the elderly assessed.

Regarding personality factors, by and large, the results showed that half of the participants had low extraversion and openness to experience scores, and high agreeableness (socialization) and conscientiousness (achievement) scores. Some research has found that individuals with PD show a lower tendency for novelty seeking, related to the factor openness to experience, compared with healthy adults.^47^^,^ 48

Individuals with low openness experience had a more conservative approach, preference for routine activities and limited interest in new activities and changes. These aspects may be more frequently found in elderly individuals, given there is a decrease in the levels of the openness factor with age.^49^ In addition, it can be inferred that, because PD is a chronic disease resulting in motor and cognitive limitations, engaging in new activities that are different from habitual ones can be difficult. This fact results in feelings of hesitation, fear and conformism, leading to low levels of openness to new experiences.

A review of the literature carried out by Santangelo et al.,^50^ found that patients with PD were described by their caregivers as rigid, introverted and cautious. This type of personality is characterized by a reduction in openness to experience, including novelty searching and avoidance behaviors.

The low levels of extraversion found in the present study sample were also identified in the study by Damholdt et al.^24^ These results may also be related to low openness levels. Thus, individuals with low extraversion have low exposure and less social stimulation, and a more reserved attitude. It is possible that more severe motor symptoms may impose greater limitations on physical mobility and social interactions. Thus, patients with PD, in addition to low interest in novelty, may have mobility difficulties and also develop feelings of shame about their own physical limitations and illness, choosing to spend more time alone and reserved.

The results of this study indicate that most patients with PD had low and average levels of neuroticism. This finding does not corroborate findings from the literature, which indicate that high levels of neuroticism are associated with PD, as well as emotional malaise and low adherence to treatment. A possible explanation for the low levels of neuroticism found is that all participants evaluated were undergoing medical monitoring and had good adherence to treatment, which may also be related to higher rates of conscientiousness and agreeableness. Thus, individuals with low levels of neuroticism and greater conscientiousness would be involved in health promotion behaviors, aiming to attain better physical health.^20^

Regarding the relationship between personality factors and cognitive functions, neuroticism correlated negatively with executive functions (abstract reasoning and problem-solving). Therefore, the higher the level of neuroticism, the lower the performance on the tasks of abstract reasoning and problem-solving. According to Kuzma et al.,^18^ high rates of neuroticism are associated with poor performance on cognitive tasks, which may reflect the effect of chronic stress on cognitive aging.

The results of this study also showed that the higher the prevalence of extraversion, the better the results for executive functions (verbal fluency). In addition, the individuals evaluated that displayed greater openness to new experiences had better performance for verbal episodic memory (delayed recall). In the study by Sutin et al.,^22^ extroverted and open participants had better results for verbal fluency. Booth et al.^26^ found similar results, observing that openness to experience had a significant relationship with levels of verbal memory and general cognitive ability. According to the authors, openness promotes lifelong behavior patterns that lead individuals to engage in learning and leisure activities.

The extraversion factor helped explain the performance of patients with PD on memory tasks (immediate recall and recognition) and in executive functions (verbal phonemic and semantic fluency). This result corroborates the findings of Sutin et al.,^22^ which indicated that high levels of extraversion and low neuroticism scores were associated with better performance of the elderly on episodic memory tasks. Together, the extraversion and openness factors helped explain the performance of the elderly with PD on verbal episodic memory (delayed recall). According to Chapman et al.,^16^ among the dimensions of the Five-factor model, the factor openness to experience is the strongest predictor for good cognitive functioning in elderly people.

The present study reports innovative data on a subject little explored in the literature. However, there are some limitations that should be noted, such as the recruitment of participants which involved a convenience sample from the Parkinson's Association of Rio Grande do Sul and an Ambulatory Movement Disorders unit of a teaching hospital. The sample was therefore homogeneous. In addition, all participants were physically and socially active in groups, which may not reflect the situation of elderly with PD in general. Another limitation was the small number of participants with PD, which prevented other types of statistical analysis.

In conclusion, it can be concluded that participants with PD exhibited impairment in the functions of verbal episodic memory (immediate recall, delayed recall and recognition) and executive functions (abstract reasoning and problem-solving). In this study, the individuals who displayed openness to new experiences had better performance for verbal episodic memory (delayed recall). Together, extraversion and openness factors proved stronger predictors of the performance of the elderly with PD on verbal episodic memory tasks (delayed recall). The extraversion factor only contributed to the performance of the elderly with PD on memory tasks (immediate recall and recognition) and executive functions (verbal phonemic and semantic fluency).

Studies involving this population focusing on the variables investigated are scarce. Therefore, further studies with longitudinal designs that include participants with PD in different contexts should be conducted to track the relationship between personality factors and cognitive functioning throughout the aging process.

## References

[1] Kalia LV, Lang AE (2015). Parkinson's disease. The Lancet.

[2] Farlow J, Pankratz ND, Wojcieszek J, Adam MP, Ardinger HH, Pagon RA (1993). Parkinson Disease Overview. 2004 May 25 [Updated 2014 Feb 27]. GeneReviews^®^.

[3] Muangpaisan W, Mathews A, Hori H, Seidel D (2011). A systematic review of the worldwide prevalence and incidence of Parkinson's disease. J Med Assoc Thai.

[4] Instituto Brasileiro de Geografia e Estatística (IBGE) (2013). Projeção da população do Brasil por sexo e idade para o período 2000/2060 e projeção da população das Unidades da Federação por sexo e idade para o período 2000/2030.

[5] Souza CFM, Almeida HCP, Sousa JB, Costa PH, Silveira YSS, Bezerra JCL (2011). A doença de Parkinson e o processo de envelhecimento motor: uma revisão de literatura. Rev Neurociênc.

[6] Flores FDT, Rossi AG, Schmidt PDS (2011). Avaliação do equilíbrio corporal na doença de Parkinson. Arq Int Otorrinolaringol.

[7] Tosin MHDS, Campos DM, Blanco L, Santana RF, Oliveira BGRBD (2015). Mapping Nursing language terms of Parkinson's disease. Rev Esc Enferm USP.

[8] Thanvi BR, Munshi SK, Vijaykumar NLOTC, Lo TCN (2003). Neuropsychiatric non-motor aspects of Parkinson's disease. Postg Med J.

[9] Radanovic M, Stella F, Forlenza OV (2015). Comprometimento cognitivo leve. Rev Med.

[10] Clark L (2015). Personality Traits in Parkinson's Disease. Undergraduate Review.

[11] Rieder CRM, Chardosim NMO, Terra NL, Gonzatti V, Argimon IIL, Chardosim NMO (2016). Entendendo a doença de Parkinson: Informações para pacientes, familiares e cuidadores.. Aspectos Cognitivos na Doença de Parkinson.

[12] Cerasa A, Gioia MC, Salsone M, Donzuso G, Chiriaco C, Realmuto S (2014). Neurofunctional correlates of attention rehabilitation in Parkinson's disease: an explorative study. Neurol Sci.

[13] Lemes LB, Batistetti CL, de Almeida IA, Barboza NM, Terra MB, Bueno MEB, Santos SMS (2016). Desempenho cognitivo-perceptual de indivíduos com doença de Parkinson submetidos à fisioterapia. ConScientiae Saúde.

[14] Janvin CC, Larsen JP, Aarsland D, Hugdahl K (2006). Subtypes of mild cognitive impairment in Parkinson's disease: progression to dementia. Mov Disord.

[15] Galhardo MMDAM, Amaral AKDFJ, Vieira ACDC (2009). Characterizing cognitive disorders in Parkinson's disease. Revista CEFAC.

[16] Chapman BP, Duberstein PR, Tindle HA, Sink KM, Robbins J, Tancredi DJ, Franks P (2012). Personality predicts cognitive function over seven years in older persons. Am J Geriatr Psychiatry.

[17] Fuentes D, Moreno C, Sassi F, Frambati L, Lacerda J, Malloy-Diniz LF, Malloy-Diniz LF, Fuentes D, Mattos P, Abreu N (2010). Avaliação da Personalidade e sua Contribuição à Avaliação Neuropsicológica. Avaliação Neuropsicológica.

[18] Kuzma E, Sattler C, Toro P, Schonknecht P, Schroder J (2011). Premorbid personality traits and their course in mild cognitive impairment: Results from a prospective population-based study in Germany. Dement Geriatr Cogn Disord.

[19] Carvalho LDF, Sette CP, Primi CGCR (2014). Propriedades psicométricas da versão revisada da dimensão necessidade de atenção do inventário dimensional clínico da personalidade. Temas Psicol.

[20] Löckenhoff CE, Terraciano A, Ferrucci L, Costa PT (2012). Five-Factor Personality Traits and Age Trajectories of Self-Rated Health: The Role of Question Framing. Journal of Personality.

[21] Fonseca A (2006). O envelhecimento: uma abordagem psicológica.

[22] Sutin AR, Terracciano A, Kitner-Triolo MH, Uda M, Schlessinger D, Zonderman AB (2011). Personality traits prospectively predict verbal fluency in a lifespan sample. Psychol Aging.

[23] Booth J, Schinka J, Brown L, Mortimer J, Borenstein A (2006). Five-factor personality dimensions, mood states, and cognitive performance in older adults. J Clin Exp Neuropsychol.

[24] Damholdt MF, Østergaard K, Borghammer P, Larsen L (2011). The parkinsonian personality and concomitant depression. J Neuropsychiatry Clin Neurosci.

[25] Costa PT, McCrae RR (2010). NEO PI-R: inventário de personalidade NEO revisado e inventário de cinco fatores NEO revisado NEO-FFI-R.

[26] Flores-Mendonza CE (2007). Inventário de personalidade NEO-revisado: manual técnico.

[27] Figueiredo VL, Do Nascimento E (2007). Desempenhos nas duas tarefas do subteste dígitos do WISC-III e do WAIS-III. Psicol Teor Pesq.

[28] Wechsler D (2004). WAIS-III: Escala de Inteligência Wechsler para Adultos: Manual; Adaptação e Padronização de uma amostra Brasileira.

[29] Memory CM, Yassuda MS, Nakano EY, Forlenza OV (2013). Brief screening for mild cognitive impairment: validation of the Brazilian version of the Montreal cognitive assessment. Int J Geriatr Psychiatry.

[30] Nasreddine S, Phillips A, Bedirian V (2005). The Montreal Cognitive Assessment, MoCA: a brief screening tool for mild cognitive impairment. J Am Geriatr Soc.

[31] Strauss E, Sherman EM, Spreen O (2006). A compendium of neuropsychological tests: Administration, norms, and commentary.

[32] Machado TH, Fichman HC, Santos EL, Carvalho VA, Fialho PP, Koenig AM (2009). Normative data for healthy elderly on the phonemic verbal fluency task-FAS. Dement Neuropsychol.

[33] Tombaugh TN, Kozak J, Rees L (1999). Normative data stratified by age and education for two measures of verbal fluency: FAS and animal naming. Arch Clin Neuropsychol.

[34] Brucki SM, Malheiros SM, Okamoto IH, Bertolucci PH (1997). Normative data on the verbal fluency test in the animal category in our milieu. Arq Neuropsiquiatr.

[35] Bertolucci PHF, Okamoto IH, Toniolo J, Ramos LR, Brucki SMD (1998). Desempenho da população brasileira na bateria neuropsicológica do Consortium to Establish a Registry for Alzheimer's Disease (CERAD). Rev Psiquiatr Clín.

[36] Rabelo IS, Pacanaro SV, Leme IFAS, Ambiel RAM, Alves GAS (2011). Teste não verbal de inteligência geral - BETA III- Subtestes raciocínio matricial e códigos. Manual Técnico.

[37] Cunha JA (2001). Manual da versão em português das Escalas Beck.

[38] Yesavage JA, Brink TL, Rose TL, Lum O, Huang V, Adey M, Leirer VO (2002). Development and Validation of a Geriatric Depression Screening Scale: a Preliminary Report. J Psychiatr Res.

[39] Shenkman ML, Clark K, Xie T, Kuchibhatla M, Shinberg M, Ray L (2001). Spinal movement and performance of standing reach task in participants with and without Parkinson disease. Phys Ther.

[40] Rocha MSG, Andrade VM, Santos FH, Bueno OFA (2004). Doença de Parkinson: aspectos neurpsicológicos.

[41] Stavitsky K, Neargarder S, Bogdanova Y, McNamara P, Cronin-Golomb A (2012). The impact of sleep quality on cognitive functioning in Parkinson's disease. J Int Neuropsychol Soc.

[42] Nuti A, Ceravolo R, Piccinni A, Dell'Agnello G., Bellini G., Gambaccini G (2004). Psychiatric comorbidity in a population of Parkinson's disease patients. Eur J Neurol.

[43] Nègre-Pagès L, Grandjean H, Lapeyre-Mestre M, Montastruc JL, Fourrier A, Lépine JP (2010). Anxious and depressive symptoms in Parkinson's disease: The French cross-sectionnal DoPaMiP study. Mov Disord.

[44] Brown RG, Landau S, Hindle JV, Playfer J, Samuel M, Wilson C (2011). Depression and anxiety related subtypes in Parkinson's disease. J Neurol Neurosurg Psychiatry.

[45] Farina M, Irigaray TQ, de Lima Argimon II (2016). Personalidade e funcionamento adaptativo e psicopatológico em idosos. Perspectivas Psicología.

[46] Koorevaar AML, Comijs HC, Dhondt ADF, Van Marwijk HWJ, Van Der Mast RC, Naarding P (2013). Big Five personality and depression diagnosis, severity and age of onset in older adults. J Affect Disord.

[47] Fujii C, Harada S, Ohkoshi N, Hayashi A, Yoshizawa K (2000). Cross-cultural traits for personality of patients with Parkinson's disease in Japan. Am J Med Genet.

[48] Rodrigues CYDS (2014). Personalidade e personalidade pré-mórbida na fase inicial da doença de Parkinson. Psique.

[49] Srivastava S, John OP, Gosling SD, Potter J (2003). Development of Personality in Early and Middle Adulthood: Set Like Plaster or Persistent Change?. J Pers Soc Psychol.

[50] Santangelo G, Piscopo F, Barone P, Vitale C (2017). Personality in Parkinson's disease: Clinical, behavioural and cognitive correlates. J Neurol Sci.

